# Quality evaluation of metabolic and bariatric surgical guidelines

**DOI:** 10.3389/fendo.2023.1118564

**Published:** 2023-03-09

**Authors:** Zi-Han Qin, Xin Yang, Ya-Qi Zheng, Li-Ya An, Ting Yang, Yu-Lu Du, Xiao Wang, Shu-Han Zhao, Hao-Han Li, Cheng-Kai Sun, Da-Li Sun, Yue-Ying Lin

**Affiliations:** ^1^ The First Affiliated Hospital of Kunming Medical University, Kunming, Yunnan, China; ^2^ Zhujiang Hospital, Southern Medical University, Guangzhou, Guangdong, China; ^3^ The Second Affiliated Hospital of Kunming Medical University, Kunming, Yunnan, China; ^4^ Department of Gastrointestinal Surgery, The Second Affiliated Hospital of Kunming Medical University, Kunming, China

**Keywords:** bariatric surgery, metabolic surgery, guidelines, recommendations, bariatric

## Abstract

**Objective:**

To evaluate the quality of surgical guidelines on bariatric/metabolic surgery.

**Methods:**

Four independent reviewers used the AGREE II (The Appraisal of Guidelines for Research and Evaluation II) tool to assess the methodological quality of the included guidelines and conducted a comparative analysis of the main recommendations for surgical methods of these guidelines.

**Results:**

Nine surgical guidelines were included in this study. Five articles with AGREE II scores over 60% are worthy of clinical recommendation. The field of rigor of development was relatively low, with an average score of 50.82%. Among 15 key recommendations and the corresponding best evidence in the guidelines, only 4 key recommendations were grade A recommendations.

**Conclusions:**

The quality of metabolic and bariatric guidelines is uneven, and there is much room for improvement.

## 1 Introduction

In recent years, the global obesity rate has increased sharply, and the obesity crisis has become one of the biggest public health challenges in the 21st century. Additionally, with the increase in disease burden, the significant increase in medical costs and indirect loss of productivity also have a huge economic impact (1, 2). For example, most adults in the United States and the United Kingdom are considered overweight from a medical perspective (body mass index [BMI] of 25 – 29.9 kg/m^2^) or obese (BMI of 30 kg/m^2^ and more) (3). With the widespread prevalence of obesity, complications such as coronary heart disease, high blood pressure, stroke, certain types of cancer, non-insulin-dependent diabetes, gallbladder disease, dyslipidaemia, osteoarthritis, gout, and sleep apnoea occur simultaneously (4, 5). Obesity and overweight can lead to shortened life expectancy and lower quality of life (6).

Although some new drugs (7) and diet and exercise programs (8) have been developed to counteract the continuous increase in the obesity rate, the above measures are still not ideal for controlling obesity. Bariatric surgery has been proven to be an effective method for the control of morbid obesity and metabolic syndrome (3) and has also received attention from international academic groups and experts on bariatric/metabolic surgery. Currently, metabolic and weight loss surgical guidelines have been developed (9–17). However, the methodological quality of these guidelines and the heterogeneity of the main recommendations have caused great confusion to the users of these guidelines. Therefore, the purpose of this study was to analyse the methodological quality of the guidelines for bariatric/metabolic surgery and the differences in recommendations between these guidelines and to provide the best evidence to help guideline users in choosing an appropriate guideline and to inform guideline developers when they update them.

## 2 Methods

### 2.1 Research design

In this study, the AGREE II tool was adopted to conduct methodological evaluation of clinical guidelines for metabolism and weight loss, and PRISMA (Preferred Reporting Items for Systematic Reviews and Meta-Analyses) principles were followed (18).

### 2.2 Retrieval strategy

In this study, metabolism and weight loss surgery-related guidelines were retrieved from PubMed, Ovid, Springer, Web of Science, CNKI, VIP database, Wanfang Database and other databases. This study examined relevant guidelines published from January 1, 2014, to January 1, 2021, that included supporting evidence for the main recommendations included in the guidelines and the impact of time span on evidence updates. This study also searched Google and Baidu Academics to obtain more guidelines. The language was limited to English and Chinese. Keywords included the Chinese and English keywords “bariatric surgery”, “metabolic surgery” and “guidelines”. Additionally, the reference lists of the included guidelines were manually searched.

### 2.3 Selection principle of guidelines

#### 2.3.1 Inclusion criteria

(1) complete guideline text (2); the guidelines include information about metabolism and bariatric surgery (3); if the guidelines are updated, only the latest version will be included (4); guidelines published in English or Chinese.

#### 2.3.2 Exclusion criteria

(1) duplicate guidelines, translated versions of the guidelines, secondary or multiple publications, and brief abstracts (2); the translated versions of the guidelines may have lost the information of the original versions, which may have affected the accuracy of the evaluation of this study (3); if multiple guidelines were issued by the same organisation, older versions of the guidelines were excluded.

According to the above inclusion and exclusion criteria, two reviewers (Xiao Wang and Yu-Lu Du) independently evaluated the obtained literature to determine whether to include or exclude the literature. Disagreements were resolved through negotiation until consensus or by consulting a third expert reviewer (Ya-Qi Zheng).

### 2.4 Quality assessment of the guidelines

#### 2.4.1 AGREE II tools

This research used the latest version of the AGREE II tool (https://www.agreetrust.org/resource-centre/) to assess each bariatric/metabolic guideline that met the standards of this study. This tool consists of 6 domains and 23 items. (**Table S1**).

In this study, each bariatric/metabolic guideline was graded according to the AGREE II user manual by four independent reviewers (Zi-Han Qin, Xin Yang, Li-Ya An and Ting Yang). The reviewers were trained on the use of the AGREE II tool through a rigorous online training course on the AGREE website. Reviewers are guided and supervised by experts (Da-Li Sun, Yue-Ying Lin, Li-Ya An and Ting Yang) who have published a number of articles through the use of AGREEII. The team includes experienced specialists in bariatric/metabolic surgery (Da-Li Sun, Yue-Ying Lin, Li-Ya An and Ting Yang). The user manual defines each item and helps the user determine the guideline score for the item. Items are rated on a scale of 1 (completely inconsistent with the item) to 7 (completely consistent with the item). Domain scores are calculated by adding up the project scores for each domain for each reviewer, then normalizing them to the percentage of the highest score (19).

For each area of the AGREE II tool, “points earned” is calculated as the sum of all points scored by the grader for all items contained in that area. The “proportional domain score” is calculated as a standardized score using the following formula: (score obtained − lowest possible score)/(maximum possible score − lowest possible score). The maximum score for each area is obtained by multiplying the number of items in that area by the number of raters, multiplying by 7 (which corresponds to “strong agreement”). The minimum score is obtained by multiplying the number of items in the field by the number of raters and multiplying by 1 (which corresponds to “strong disagreement”).

### 2.5 Guidelines for extracting and regrading key recommendations and best evidence

In this study, the relatively high AGREE II scoring guidelines were used to extract and analyse important recommendations related to metabolism and bariatric surgery, and a database search was conducted to further obtain the highest level of evidence supporting these recommendations. This study reclassified recommendations and evidence using the Oxford Centre for Evidence-based Medicine (OCEBM) grading system. (**Table S2**).

### 2.6 Statistical analysis

We used a descriptive statistical analysis method to calculate the standardized scores for each guideline, which were expressed as a percentage, and we also listed the median scores and the range of each domain. We adopted a two-way ANOVA to calculate the intra-class correlation coefficients (ICCs) to examine the agreement among the scores from the four reviewers. Consistency among raters was determined by ICCs and 95% confidence intervals (CIs). ICC is equal to the individual variation divided by the total variation, with a value between 0 and 1 for the 23 items identified in AGREE II. If the ICC is between 0.01 and 0.20, the degree of agreement is considered to be slight; if the ICC is between 0.21 and 0.40, the consistency is considered fair. If the ICC is between 0.41 and 0.60, the consistency is moderate. If the ICC is between 0.61-0.80, the degree of agreement is considered to be high; if the ICC is between 0.81-1.00, it is considered perfect. P < 0.05 indicates statistical significance. Statistical analysis was performed using IBM SPSS Version 19.0 (SPSS Inc., Chicago, IL, USA). (**Table S3**).

## 3 Results

### 3.1 Guideline features

**Figure 1:**
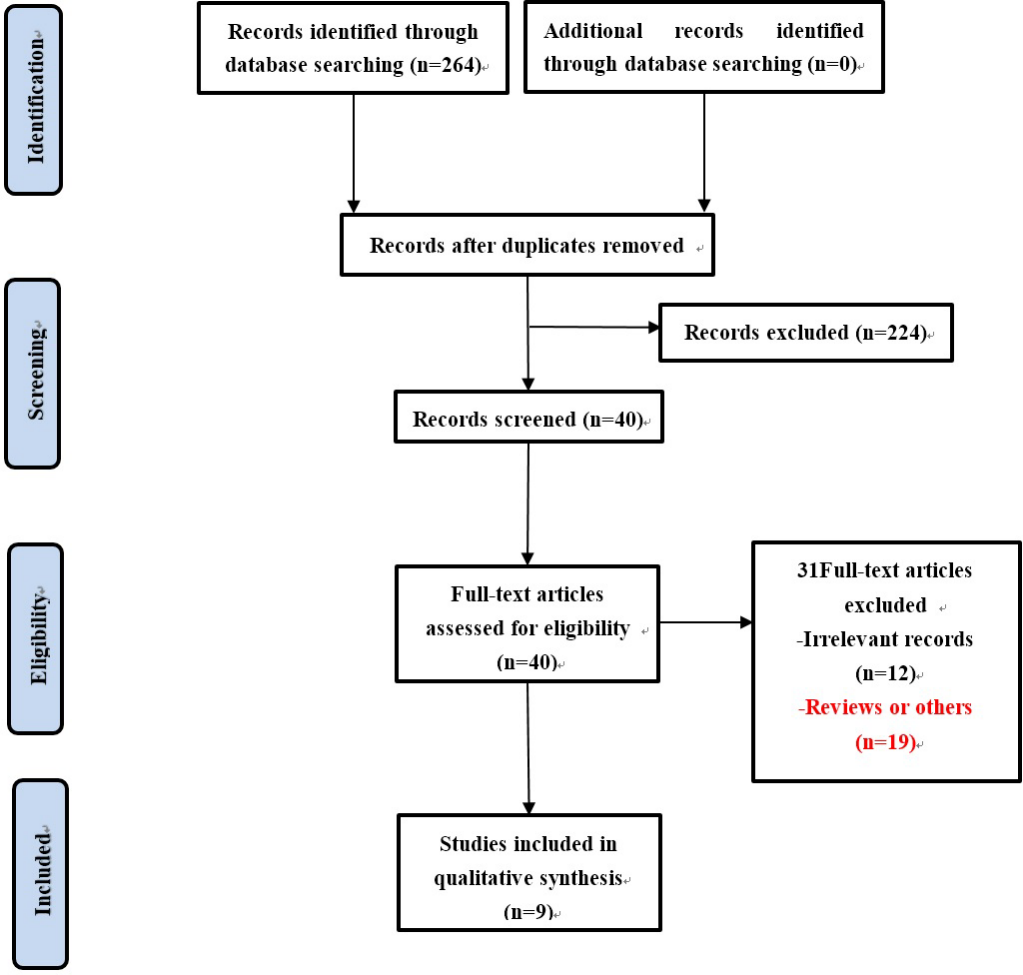
Flowchart of study selection.

A total of 264 records were obtained through database retrieval and other retrieval methods, which were evaluated by reading titles, abstracts and full texts. Finally, 9 guidelines for bariatric/metabolic surgery were included (**Figure 1**), 8 of which are original (9–14, 16, 17) and 1 of which was updated in 2020 (15). All guidelines were developed by local or national medical associations. Seven of the guidelines are international or from > 1 country (9–14, 16), one is from the EU (15), and one is from India (17). One of the nine guidelines is for children and adolescents (10), and the other eight are for adults (9, 11–17). The basic features of the included guidelines are listed in **Table 1**.

**Table 1:** Characteristics of included guidelines.

Title	Authors	Organization	Short name	Country Applied	Grading system	Subjects	Version	Target population	Development Method
Reoperative surgery for nonresponders and complicated sleeve gastrectomy operations in patients with severe obesity. An international expert panel consensus statement to define best practice guideline	Kichler K, et al.,2018	ASMBS	Ki (9)	International	Not specified	Providing a clinical consensus guideline regarding standardization of indications, contraindications, surgical options, and surgical techniques when reoperating on patients who underwent a failed or complicated SG.	Original version	Adults	CB
ASMBS pediatric metabolic and bariatric surgery guidelines	Pratt JSA, et al.,2018	ASMBS	Pr (10)	International	Not specified	Removing the stigma against the surgical treatment of childhood obesity and educate paediatric physicians and providers about the need for early referral of patients suffering from severe obesity to a MBS program.	Original version	Children & Adolescents	EB
The first consensus statement on revisional bariatric surgery using a modified Delphi approach	Mahawar KK, et al.,2019	IFSO	Ma (11)	International	Not specified	Developing consensus amongst a group of international RBS experts on a range of practices and principles concerning this procedure following a Modified Delphi protocol.	Original version	Adults	CB
Bariatric surgery in class I obesity: a Position Statement from the International Federation for the Surgery of Obesity and Metabolic Disorders (IFSO)	Busetto L, et al.,2014	IFSO	Bu (12)	International	Not specified	Examining the use of bariatric surgery in the class I obesity range (BMI 30 - 35 kg/m^2^).	Original version	Adults	EB & CB
Duodenal switch in revisional bariatric surgery: conclusions from an expert consensus panel	Merz AE, et al.,2019	ASMBS	Me (13)	International	Not specified	Generating expert consensus points on the appropriate use of BPD/DS in the revisional bariatric surgical setting	Original version	Adults	EB & CB
ASMBS Updated Position Statement on Bariatric Surgery in Class I Obesity (BMI 30-35 kg/m^2^)	Aminian A, et al.,2018	ASMBS	Am (14)	International	Not specified	Assessing the evidence regarding the benefits and risks of bariatric surgery in patients with class I obesity (BMI of 30.0 -- 34.9 kg/m^2^), which accounts for more than 20% of the United States population	Original version	Adults	EB
Clinical practice guidelines of the European Association for Endoscopic Surgery (EAES) on bariatric surgery: update 2020 endorsed by IFSO-EC, EASO and ESPCOP	Lorenzo ND, et al.,2020	EAES	Lo (15)	Europe	GRADE	Aiming to increase health care knowledge among bariatric patients. Summarizing the latest evidence on bariatric surgery through state-of-the art guideline development, aiming to facilitate evidence-based clinical decisions	Updated version	Adults	EB & CB
IFSO (International Federation for Surgery of Obesity and Metabolic Disorders) Consensus Conference Statement on One-Anastomosis Gastric Bypass (OAGB-MGB): Results of a Modified Delphi Study	Ramos AC, et al.,2020	IFSO	Ra (16)	International	Not specified	Validating the results of the previous exercise as well as to expand into areas not previously covered.	original version	Adults	EB & CB
OSSI (Obesity and Metabolic Surgery Society of India) Guidelines for Patient and Procedure Selection for Bariatric and Metabolic Surgery	Bhasker AG, et al.,2020	OSSI	Bh (17)	India	Not specified	Enlisting the OSSI guidelines for patient and procedure selection for surgeons and allied health practitioners practising bariatric and metabolic surgery. Intending to guide Insurance Regulatory and Development Authority of India and multiple other stake-holders.	original version	Adults	EB & CB

EB, Guidelines based on evidence-based medicine; CB, Develop guidelines based on expert consensus; ASMBS, American Society for Metabolic and Bariatric Surgery; IFSO, International Federation for Surgery of Obesity and Metabolic Diseases; EAES, European Association of Endoscopic Surgery; EASO, European Association for the Study of Obesity; ESPCOP, European Society for the Peri-operative Care of the Obese Patient; OSSI, Obesity and Metabolic Surgery Society of India; SG, Sleeve gastrectomy; MBS, Metabolic and bariatric surgery; RBS, Revisional bariatric surgery; BMI, Body mass index; BPD/DS, Biliopancreatic diversion with duodenal switch; GRADE, Grade of recommendations assessment, development and evaluation.

### 3.2 Quality evaluation of the guidelines

The AGREE II standardized area scores for each metabolic and bariatric/metabolic surgery recommendation guideline and their overall recommendations are shown in **Table 2**. Domain scope and purpose and domain clarity and presentation methods had the highest median scores of 84.09% and 76.70%, respectively (range 77.78% to 91.67% and 62.50% to 91.67%, respectively). The median score for domain stakeholder involvement was 60.03% (range 47.22% to 86.11%). Only one guideline (11%) scored less than 50% (13). Editorial independence of the domain had the highest score range (0 to 100%). Two guidelines scored zero (9, 12), and three guidelines scored less than 50% (9, 10, 12). The median scores for applicability and rigor of development were 45.95% and 50.82% (ranging from 27.08% to 78.13% and 20.83% to 77.08%, respectively). According to the overall score of the guidelines, the five included guidelines scored well in all areas (10–12, 14, 15) and were classified as strongly recommended for clinical practice. Four guidelines were recommended for revisions (9, 13, 16, 17).

**Table 2:** AGREE II domain score and ICC of the included guidelines.

Guidelines	Scope and Purpose	Stakeholder Involvement	Rigour of Development	Clarity and Presentation	Applicability	Editorial independence	Overall assessment	ICC
Ki (9)	77.78%	50.00%	20.83%	79.17%	27.08%	0.00%	37.85%	0.947
Pr (10)	91.67%	86.11%	70.83%	77.78%	77.08%	47.92%	74.91%	0.881
Ma (11)	77.79%	52.78%	48.44%	77.78%	54.17%	93.75%	63.42%	0.892
Bu (12)	87.50%	52.78%	77.08%	66.67%	78.13%	0.00%	64.67%	0.905
Me (13)	83.33%	47.22%	56.25%	87.50%	46.88%	50.00%	59.29%	0.915
Am (14)	80.56%	79.17%	55.21%	84.72%	33.33%	100.00%	65.19%	0.837
Lo (15)	91.67%	69.44%	68.32%	91.67%	55.21%	50.00%	68.73%	0.913
Ra (16)	83.22%	51.39%	30.21%	62.50%	20.83%	50.00%	43.65%	0.897
Bh (17)	83.33%	51.39%	30.21%	62.50%	20.83%	50.00%	43.66%	0.922
Median score (range)	84.09% (77.78%-91.67%)	60.03% (47.22%-86.11%)	50.82% (20.83%-77.08%)	76.70% (62.5%-91.67%)	45.95% (27.08%-78.13%)	49.07% (0%-100%)	57.93% (37.85%-68.73%)	

ICC, Intraclass correlation coefficient.

In this study, four evaluators participated in the evaluation of the guidelines, and the ICC value range of the metabolic and bariatric surgery guidelines was > 0.8 using AGREE II, indicating a high degree of internal item score consistency among reviewers.

### 3.3 Key recommendations in the guidelines and the best available evidence

To further analyse the reasons for the heterogeneity of recommendations for metabolism and bariatric surgery among different guidelines, this study referred to guidelines (15) with relatively high scores and relatively clear recommendation items, extracted the main recommendations in the guidelines, and sorted out the highest evidence supporting these main recommendations by searching the database. Additionally, recommendations (9–11, 14, 16) not included in the high quality guideline (15) are also sorted (**Table 3**). It mainly includes surgical indications, surgical methods, and preoperative and postoperative recommendations (**Table 3**).

**Table 3:** The key recommendations and the best evidence to support the recommendations at present.

	The key recommendations	The best evidence to support the recommendations at present	Strength of recommendation	Quality of evidence	Ki (9)	Pr (10)	Ma (11)	Bu (12)	Me (13)	Am (14)	Lo (15)	Ra (16)	Bh (17)
Indications of bariatric/metabolic surgery	Bariatric/metabolic surgery should be considered for patients with BMI≥ 35 kg/m2 with associated comorbidities	A RCT including 57 patients (20).	B	2b	1	1	1	1	1	1	3	1	3
	Bariatric/metabolic surgery should be considered for patients with BMI≥ 30 kg/m2 and type 2 diabetes with poor control despite optimal medical therapy	A systematic review of 11 RCTs (21)	A	1a	1	1	1	1	1	3	3	3	3
	Bariatric/metabolic surgery should be considered for patients with BMI≥ 30 kg/m2 and obesity-related comorbidities that cannot lose enough weight through nonsurgical treatment	A RCT including 80 patients (22).	B	2b	1	1	1	3	1	3	1	1	1
Operative methods of bariatric/metabolic surgery	SG should be preferred over AGB	A cohort study including 71 patients (23).	B	2b	3	2	1	1	1	1	2	1	1
	RYGB should be preferred over AGB	A cohort study including 1295 patients (24).	B	2b	1	2	1	1	1	1	3	1	1
	OAGB may offer greater short-term weight loss than SG	A cohort study including 123 patients (25).	B	2b	1	1	1	1	1	1	3	3	1
	OAGB may offer greater short-term weight loss than RYGB	A RCT including 253 patients (26).	A	1b	1	1	1	1	1	1	3	3	1
	RYGB is an acceptable revisional bariatric surgery option after AGB	A case series analysis including 58 patients (27).	C	4	1	2	3	1	1	1	1	1	1
	BPD/DS is an acceptable revisional bariatric surgery option after SG	A case series analysis including 33 patients (28).	C	4	3	1	3	1	3	1	1	1	1
	SADIs is an acceptable revisional bariatric surgery option after SG	A case series analysis including 63 patients (29).	C	4	3	1	3	1	3	1	1	1	1
	BPD/DS is a more acceptable revisional bariatric surgery option than RYGB after SG	A cohort study including 74 patients (28).	C	4	3	1	1	1	3	1	1	1	1
	RYGB is an acceptable surgery option for patients with gastroesophageal reflux disease after SG	A case series analysis including 10 patients( 30)	C	4	3	1	1	1	3	1	1	1	1
Preoperative work-up	Preoperative nutritional assessment can be considered before bariatric/metabolic surgery	A RCT including 120 patients (31).	A	1b	1	2	3	1	1	1	3	1	1
	Psychological evaluation can be considered before bariatric/metabolic surgery	A cohort study including 2458 patients (32).	B	2b	1	1	3	1	1	1	2	1	1
Postoperative care	Micro and/or macronutrients supplementation is recommended after bariatric/metabolic surgery	A systematic review of 5 RCTs and 7 observational studies (33).	B	2a	1	2	1	1	1	3	3	1	1
	Postoperative behavioural advice should be provided to patients undergoing bariatric/metabolic surgery	A RCT including 144 patients (34).	A	1b	1	2	1	1	1	1	3	1	1
	Pregnancy after bariatric/metabolic surgery should be delayed during the weight loss phase	Expert opinion (15).	D	5	1	2	1	1	1	1	3	1	3

BMI, Body mass index; RCT,Randomized controlled trial; SG, Sleeve gastrectomy; AGB, Adjustable gastric banding; RYGB,Roux-en-Y gastric bypass; OAGB, One anastomosis gastric bypass; BPD/DS, Biliopancreatic diversion with duodenal switch; SADIs, Single anastomosis duodeno-ileal bypass; indicates being recommended definitely (including position statements and agreement of consensus) = 3; indicates being mentioned or conditionally recommended = 2; indicates being not mentioned = 1.

## 4 Discussion

Compared to other disease-specific guidelines, the development of guidelines for metabolism and bariatric surgery can be a complex issue, as metabolism and bariatric surgery is a multidisciplinary, global issue. In this study, even within the same guidelines, the quality of metabolic and bariatric surgery guidelines was highly heterogeneous among different fields, and there were significant differences in the distribution of evidence level and recommendation strength between different categories of guidelines.

Analysing the included guidelines, the study identified several areas where improvement was needed in the development of the guidelines. In the development of guidelines, patient perceptions, expectations and preferences for medical care have become increasingly important. Stakeholder participation can well reflect the views of prospective users and patients. The implementation of the guidelines also requires multidisciplinary medical expertise. However, the guidelines included in this study did not provide details about the involvement of patients and their representatives.

Rigour of development is closely related to credibility in the implementation of the guidelines. This field assessment is used to locate and synthesize evidence and to develop and update recommendations (35). Unsystematic methods of retrieving evidence tend to lead to low-quality guidelines (11, 16, 17). The lack of clear evidence selection criteria and their strengths and limitations (9, 16) also lead to the low quality of the guidelines. Other causes include vague connection between recommendations and evidence, lack of external evaluation, failure to provide guideline update steps (9, 11, 16), and failure to consider side effects and risks when forming recommendations (17).

All nine guidelines included in the study scored > 60% for clarity and presentation. This shows that the recommendations in the guidelines are clear and easy to identify.

The overall score for the applicability of the guidelines was low in the included guidelines. This indicates that the hindrance and promotion factors in the application of the guidelines have not been fully understood in the formulation of the guidelines (16). No recommendations or tools are provided in the application to ensure its feasibility (9, 17). Additionally, the neglect of relevant resources (9, 11, 12, 14–17) that may be needed in the application of recommendations and the lack of monitoring and auditing standards (9, 11, 13, 14, 17) are also important reasons for the low score of the guidelines in the application field.

In the area of editorial independence of the guideline. The influence of sponsors’ views on the guideline and the conflicts of interest between members of the organisations involved in the development of the guideline are rarely mentioned (9, 12). Therefore, conflicts of interest among members should be clearly recorded and publicized in the formulation of the guidelines to improve their independence.

There are significant differences in recommendations in the included guidelines. Therefore, this study further analysed the consistency and controversy between current recommendations and corresponding evidence of metabolic and bariatric surgery guidelines with reference to key recommendations from the guidelines for metabolic and bariatric surgery with relatively high scores.

### 4.1 Indications for bariatric/metabolic surgery

#### 4.1.1 (1) Bariatric/metabolic surgery should be considered for patients with BMI≥ 35 kg/m^2^ with associated comorbidities (recommendation strength: B; evidence level: 2b) (20)

For this recommendation, only 2 guidelines (15, 17) are relatively consistent in this recommendation, and the other 7 guidelines (9–14, 16) do not specify this recommendation. However, there is currently a lack high-quality empirical evidence, and the best evidence derives from a randomized controlled trial of 57 patients. The main conclusion is that surgery is very effective in the short term for patients with T2DM and obesity (20).

#### 4.1.2 (2) Bariatric/metabolic surgery should be considered for patients with BMI≥ 30 kg/m^2^ and type 2 diabetes with poor control despite optimal medical therapy (recommendation strength: A; evidence level: 1a) (21)

For this recommendation, four guidelines (14–17) are relatively consistent in this regard, while the other five guidelines (9–13) do not explicitly state this recommendation. The best evidence to date is a systematic review of 11 randomized controlled trials, with the main conclusion that bariatric/metabolic surgery is more effective than various medical/lifestyle interventions in reducing body weight, controlling blood glucose, alleviating T2DM, and improving other cardiovascular disease risk factors (21).

#### 4.1.3 (3) Bariatric/metabolic surgery should be considered for patients with BMI≥ 30 kg/m^2^ and obesity-related comorbidities that cannot lose enough weight through nonsurgical treatment (recommendation strength: B; evidence level: 2b) (22)

For this recommendation, two guidelines (12, 14) are relatively consistent in this regard, while the other seven guidelines (9–11, 13, 15–17) do not explicitly state this recommendation. There is a lack of high-quality evidence from a large sample. The best available evidence is a randomized controlled trial involving 80 patients who shows that surgical treatment was statistically significant compared with nonsurgical treatment in terms of weight loss, solving metabolic syndrome and improving quality of life for adults with mild to moderate obesity (body mass index, 30 kg/m^2^ to 35 kg/m^2^) (22).

### 4.2 Operative methods of bariatric/metabolic surgery

#### 4.2.1 (1) SG should be preferred over AGB (recommendation strength: B; evidence level: 2b) (23)

Three guidelines [9, 10 and 15] agree on this recommendation. The other six guidelines (11–14, 16, 17) do not specify this recommendation. There is still a lack of high-quality research evidence with a large sample, and the best evidence at present is a cohort study of 71 patients, which shows that AGB surgery is inferior to SG surgery in weight loss (23).

#### 4.2.2 (2) RYGB should be preferred over AGB (recommendation strength: B; evidence level: 2b) (24)

Two guidelines (10, 15) agree on this recommendation. The other seven guidelines (9, 11–14, 16, 17) do not explicitly state this recommendation. Currently, there is a lack of large-scale, high-quality randomized controlled studies, and the best evidence is based on a cohort study of 1295 patients in whom RYGB has a lower incidence of long-term complications than AGB (24).

#### 4.2.3 (3) OAGB may offer greater short-term weight loss than SG (recommendation strength: B; evidence level: 2b) (25)

For this recommendation, two guidelines (15, 16) have agreed on this recommendation. The other seven guidelines (9–14, 17) do not specify this recommendation. Currently, there is a lack of high-quality research evidence, and the best evidence is a cohort study involving 123 patients. The main conclusion shows that the two surgery methods have excellent weight loss and maintenance effects in the short and medium term, and the results of T2D and HTN after OAGB are better (25).

#### 4.2.4 (4) OAGB may offer greater short-term weight loss than RYGB (recommendation strength: A; evidence level: 1b) (26)

For this recommendation, two guidelines (15, 16) have agreed on this recommendation. The other seven guidelines (9–14, 17) do not specify this recommendation. The best evidence to date is a 253-patient randomized controlled trial that shows that the OAGB group has better short-term weight loss (26).

#### 4.2.5 (5) RYGB is an acceptable revisional bariatric surgery option after AGB (recommendation strength: C; evidence level: 4) (27)

Two guidelines (10, 11) agree on this recommendation. There is currently a lack of related high-quality RCT studies, and the best evidence thus far comes from a case series analysis of 58 patients, which found that RYGB is a safe operation with good weight loss within 5 years. It can be regarded as a good revision operation after failure of AGB (27).

#### 4.2.6 (6) BPD/DS and SADIs are acceptable revisional bariatric surgery options after SG (recommendation strength: C; evidence level: 4) (28, 29)

The best evidence to date comes from a case-analysis study of 96 patients, which found that BPD/DS is a safe and effective option after initial SG failure, especially in patients with severe obesity before SG. SADI-S results in a more significant reduction in overall weight than RYGB after failure of SG (28, 29).

#### 4.2.7 (7) RYGB and BPD/DS are acceptable surgical options for patients with GERD after SG surgery, and BPD/DS is better than RYGB (recommendation strength: C; evidence level: 4) (28, 30)

Two guidelines (9, 13) agree on this recommendation, and the best evidence thus far comes from a case study of 10 patients. The main conclusion is that RYGB is an effective treatment for BE and reflux after SG, and RYGB alleviates BE and reflux in most cases (30).

### 4.3 Preoperative work-up

#### 4.3.1 (1) Preoperative nutritional assessment can be considered before bariatric/metabolic surgery (recommendation strength: A; evidence level: 1b) (31)

Four guidelines (9–11, 15) have consistent opinions on this article. The other five guidelines (12–14, 16, 17) do not specify this recommendation. At present, the best evidence comes from a randomized controlled study involving 120 obese patients (31), and the results suggest that proper nutritional assessment and preoperative preparation of a balanced energy diet for morbidly obese patients can reduce the risk of surgery and improve efficacy.

#### 4.3.2 (2) Psychological evaluation can be considered before bariatric/metabolic surgery (recommendation strength: B; evidence level: 2b) (32)

Two guidelines (11, 15) recommend psychological assessment. The other seven guidelines (9, 10, 12–14, 16, 17) do not explicitly state this recommendation. The best evidence to date comes from a cohort study of 2,458 patients, which showed that measures such as preoperative psychological evaluation can improve the prevalence of alcohol use disorder after bariatric/metabolic surgery (32).

### 4.4 Postoperative care

#### 4.4.1 (1) Micro- and/or micronutrient supplementation is recommended after bariatric/metabolic surgery (recommended intensity: B; evidence level: 2a) (33)

This recommendation is relatively consistent in most guidelines (10, 13–15), while the other five guidelines (9, 11, 12, 16, 17) do not specify this recommendation. The best evidence to date includes a meta-analysis of 5 randomized controlled trials and 7 observational studies, showing that daily nutrient supplementation can effectively prevent postoperative complications (33).

#### 4.4.2 (2) Postoperative behavioural advice should be provided to patients undergoing bariatric/metabolic surgery (recommendation strength: A; evidence level: 1b) (34)

For this proposal, two guidelines (10, 15) are consistent. The guidelines recommend providing motivation intervention for postoperative patients through a randomized controlled trial of 144 obese patients. The result indicates that participants accept behavioural intervention based on the scores on the Beck depression rating scale being significantly lower than those among standard treatment participants (34).

#### 4.4.3 (3) Pregnancy after bariatric/metabolic surgery should be delayed during the weight loss phase (recommendation strength: D; evidence level: 5) (15)

Three guidelines (10, 15, 17) are consistent in this recommendation. At present, the best evidence comes from expert opinions (15), with a low level of evidence and a lack of high-quality randomized controlled studies.

In summary, suggestions for improving the quality of metabolic and bariatric surgery guidelines are as follows: (1) Developers should set different groups (including patients and the public, etc.) clearly and fully consider the views and wishes of target groups when formulating clinical guidelines. (2) In the formulation, evidence standards should be clearly described, the link between recommendations and evidence should be shown clearly, and update steps should be provided. (3) Guideline developers should be familiar with guideline development standards, such as the AGREE II tool. (4) The guideline shall be externally reviewed by experts before publication. (5) Most of the key recommendations for metabolic and bariatric surgery are not supported by high-quality research evidence. It is recommended that international academic groups on metabolic and bariatric surgery organize and carry out multicentre high-quality research to provide high-quality evidence for the key recommendations on metabolic and bariatric surgery.

This study has some advantages and limitations.

The advantages of this study are as follows: (1) This study collated and analysed the key recommendations and relevant evidence in the recent guidelines for metabolic and bariatric surgery. This study identified issues of recommendations and evidence related to metabolic and bariatric surgery and suggests improvements that may help guideline makers and users identify gaps in practice and provide a reference for guideline users to select more reliable guidelines. (2) Most of the developers of the guidelines included in this study come from international organisations and from different backgrounds, including clinical experts and methodologists, who have rich experience in developing clinical guidelines, which improves the reliability of the results of this study.

The limitations of this study are as follows: (1) In this study, only the guidelines written in English were evaluated, and the guidelines published in other languages may be missed in this study, resulting in inadequate representation of some less developed countries. (2) AGREE II is a methodological tool that does not evaluate the content and clinical significance of guidelines and focuses mainly on the formulation of guidelines. Thus, even if the guidelines are based on low-quality evidence, but the methodology is developed in compliance with AGREE II standards, the guidelines may still be scored highly by the AGREE II tool. (3) We decided to include all populations without setting age limits, but only one remaining article deals with the population of children and adolescents. This may be due to the lack of literatures and guidelines.

## 5 Conclusion

The quality of metabolic and bariatric surgical guidelines varies visibly. High-quality guidelines require multidisciplinary collaboration. Using the AGREE II tool, this study found significant room for improvement in the guidelines for metabolic and bariatric surgery, especially in terms of rigor, stakeholders, adaptability, and independence of guideline development. Effectively addressing these issues has vital implications for developing high-quality recommendations for metabolic and bariatric surgery guidelines.

## Author contributions

Z-HQ and XY were responsible for the information retrieval and the summary of characteristics of included guidelines; Y-LD and XW independently evaluated the obtained literature to determine whether to include or exclude the literature; Z-HQ, XY, L-YA, and TY were responsible for grading the included guidelines according to the AGREE II user manual; XY was responsible for writing, edit and revision of the body text; Z-HQ was responsible for the summary of key recommendations of included guidelines and corresponding best supportive evidences, and calculation of ICCs; Z-HQ, XY, and D-LS were drafted and revised the article; Z-HQ, XY, D-LS, and Y-YL were responsible for the theme, final editing, and preparation of the manuscript for submission; D-LS and Y-YL critically revised the manuscript. All authors contributed to the article and approved the submitted version.

## References

[1] Robinson E Haynes A Sutin A Daly M. Self-perception of overweight and obesity: A review of mental and physical health outcomes. Obes Sci Pract (2020) 6(5):552–61. doi: 10.1002/osp4.424 PMC7556430 33082997

[2] Imes CC Burke LE. The obesity epidemic: The USA as a cautionary tale for the rest of the world. Curr Epidemiol Rep (2014) 1:82–8. doi: 10.1007/s40471-014-0012-6 PMC4066984 24977112

[3] Fátima SP Eduardo DL Ainitze I María SM Víctor VA Amador GR. Quality criteria in bariatric surgery: Consensus review and recommendations of the Spanish association of surgeons and the Spanish society of bariatric surgery. Cir Esp (2017) 95(1):4–16. doi: 10.1016/j.ciresp.2016.09.007 27979315

[4] Obesity: preventing and managing the global epidemic. report of a WHO consultation. World Health Organ Tech Rep Ser (2000) 894:1–253. doi: 10.1177/1535370216688567 11234459

[5] Corbin LJ Richmond RC Wade KH. Body mass index as a modifiable risk factor for type 2 diabetes: Refining and understanding causal estimates using mendelian randomisation. PMC (2016) 65(10):3002–7. 10.2337/db16-0418 PMC5279886 27402723

[6] Lespessailles E Toumi H. Vitamin d alteration associated with obesity and bariatric surgery. Exp Biol Med (2017) 242(10):1086–94. doi: 10.1177/1535370216688567 PMC5444639 28103699

[7] Ali MR Moustarah F Kim JJ. American Society for metabolic and bariatric surgery position statement on intra-gastric balloon therapy. ScienceDirect (2016) 12(3):462–7. 10.1016/j.soard.2015.12.026 27056407

[8] Fátima SP Eduardo D-AL Ainitze I Socas Macias M Valentí Azcárate V García Ruiz de Gordejuela A. (2017). Quality Criteria in Bariatric Surgery: Consensus Review And Recommendations of the Spanish Association of Surgeons and the Spanish Society of Bariatric Surgery. Cir Esp. 95(1):4–16. 27979315 10.1016/j.ciresp.2016.09.007

[9] Kichler K Raul J R. Re-operative surgery for non-responders and complicated sleeve gastrectomy operations in patients with severe obesity. an international expert panel consensus statement to defifine best practice guidelines. Surg Obes Related Diseases (2019) 15(2):173–86. doi: 10.1016/j.soard.2018.11.006 31010649

[10] Janey SA Allen B Nancy TB Matias B Megan C Ashish D. ASMBS pediatric metabolic and bariatric surgery guidelines, 2018. Surg Obes Relat Dis (2018) 14(7):882–901. doi: 10.1016/j.soard.2018.03.019 30077361 PMC6097871

[11] Kamal KM Jacques MH Scott AS Almino CR Antonio T Shaw S. The first consensus statement on revisional bariatric surgery using a modified Delphi approach. Surg Endosc (2020) 34(4):1648–57. doi: 10.1007/s00464-019-06937-1 31218425

[12] Luca B John D Maurizio D Scott S Walter P Luigi A. Bariatric surgery in class I obesity: a position statement from the international federation for the surgery of obesity and metabolic disorders (IFSO). Obes Surg (2014) 24(4):487–519. doi: 10.1007/s11695-014-1214-1 24638958

[13] Alexa EM Robin BB Michel G Antonio JT Jacques H Kelvin DH. Duodenal switch in revisional bariatric surgery: conclusions from an expert consensus panel. Surg Obes Relat Dis (2019) 15(6):894–9. doi: 10.1016/j.soard.2019.03.009 31076367

[14] Aminian A Chang J Brethauer SA Kim JJ. ASMBS updated position statement on bariatric surgery in class I obesity (BMI 30-35 kg/m 2). Surg Obes Relat Dis (2018) 14(8):1071–87. doi: 10.1016/j.soard.2018.05.025 30061070

[15] Nicola Di L Stavros AA Rachel LB Luca B. Clinical practice guidelines of the European association for endoscopic surgery (EAES) on bariatric surgery: Update 2020 endorsed by IFSO-EC, EASO and ESPCOP. Surg Endosc (2020) 34(6):2332–58. doi: 10.1007/s00464-020-07555-y PMC7214495 32328827

[16] Almino CR Jean-Marc C Kamal M Wendy B Lilian K Kevin PW. IFSO (International federation for surgery of obesity and metabolic disorders) consensus conference statement on one-anastomosis gastric bypass (OAGB-MGB): Results of a modified Delphi study. Obes Surg (2020) 30(5):1625–34. doi: 10.1007/s11695-020-04519-y 32152841

[17] Bhasker AG Prasad A P Praveen Raj OSSI. (Obesity and metabolic surgery society of India) guidelines for patient and procedure selection for bariatric and metabolic surgery. Obes Surg (2020) 30(6):2362–8. doi: 10.1007/s11695-020-04497-1 32125645

[18] Wang-Qin S Yao L Wang X-q. Quality assessment of cancer cachexia clinical practice guidelines. Cancer Treat Rev Epub (2018) 70:9–15. 10.1016/j.ctrv.2018.07.008 30053727

[19] Plodkowski RA St Jeor ST. Medical nutrition therapy for the treatment of obesity. Endocrinol Metab Clin North Am (2003) 32(4):935–65. doi: 10.1016/S0889-8529(03)00077-X 14711069

[20] Manish P Chung M Sheth S. Randomized pilot trial of bariatric surgery versus intensive medical weight management on diabetes remission in type 2 diabetic patients who do NOT meet NIH criteria for surgery and the role of soluble RAGE as a novel biomarker of success. Ann Surg (2014) 260(4):617–22. doi: 10.1097/SLA.0000000000000919 PMC4691842 25203878

[21] David EC Cohen RV. Bariatric/Metabolic surgery to treat type 2 diabetes in patients with a BMI <35 kg/m2. Diabetes Care (2016) 39(6):924–33. doi: 10.2337/dc16-0350 PMC4878219 27222550

[22] O’Brien PE Dixon JB Laurie C Skinner S Proietto J McNeil J. Treatment of mild to moderate obesity with laparoscopic adjustable gastric banding or an intensive medical program: A randomized trial. Ann Intern Med (2006) 144(9):625–33. doi: 10.7326/0003-4819-144-9-200605020-00005 16670131

[23] Strain GW Michel G Pomp A Gregory D William B I Jane H. Comparison of weight loss and body composition changes with four surgical procedures. Surg Obes Relat Dis (2009) 5(5):582–7. doi: 10.1016/j.soard.2009.04.001 19560983

[24] Nguyen NQ Philip G Justin B Tamara LD Melissa N Carly MB. Outcomes of roux-en-Y gastric bypass and laparoscopic adjustable gastric banding. World J Gastroenterol (2013) 19(36):6035–43. doi: 10.3748/wjg.v19.i36.6035 PMC3785625 24106404

[25] Prashant S Susmit K Mathias F Mahak B Manoj R Rajat G. Banded sleeve gastrectomy and one anastomosis gastric Bypass/Mini-gastric bypass for treatment of obesity: A retrospective cohort comparative study with 6 years follow-up. Obes Surg (2020) 30(4):1303–9. doi: 10.1007/s11695-019-04369-3 31898044

[26] Maud R Philippe E Elise P Robert C Adrien S Lita K. Efficacy and safety of one anastomosis gastric bypass versus roux-en-Y gastric bypass for obesity (YOMEGA): A multicentre, randomised, open-label, non-inferiority trial. Lancet (2019) 393(10178):1299–309. doi: 10.1016/S0140-6736(19)30475-1 30851879

[27] Dakour Aridi HN Wehbe M-R Shamseddine G Ramzi SA Bassem YS. Long-term outcomes of roux-en-Y gastric bypass conversion of failed laparoscopic gastric band. Obes Surg (2017) 27(6):1401–8. doi: 10.1007/s11695-016-2529-x 28108969

[28] Andalib A Hussam A Almuhanna Y Bouchar P Demyttenaere S Court O. Short-term outcomes of revisional surgery after sleeve gastrectomy: a comparative analysis of re-sleeve, roux en-y gastric bypass, duodenal switch (Roux en-y and single-anastomosis). Surg Endosc (2021) 35(8):4644–52. doi: 10.1007/s00464-020-07891-z 32780238

[29] Phillip JD May Al N Laura H Eric JH Dingeman JS René MJW. Single anastomosis duodenoileal bypass or roux-en-Y gastric bypass after failed sleeve gastrectomy: Medium-term outcomes. Obes Surg (2021) 31(11):4708–16. doi: 10.1007/s11695-021-05609-1 PMC8490218 34398380

[30] Felsenreich DM Langer FB Bichler C Magdalena E Julia J Ivan K. Roux-en-Y gastric bypass as a treatment for barrett’s esophagus after sleeve gastrectomy. Obes Surg (2020) 30(4):1273–9. doi: 10.1007/s11695-019-04292-7 31808119

[31] Carbajo MA Castro MJ Kleinfinger S Gómez-Arenas S Ortiz-Solórzano J Wellman R. Effects of a balanced energy and high protein formula diet (Vegestart complet^®^) vs. low-calorie regular diet in morbid obese patients prior to bariatric surgery (laparoscopic single anastomosis gastric bypass): A prospective, double-blind randomized study. Nutr Hosp (2010) 25(6):939–48. 21519764

[32] King WC Chen J-Y Mitchell JE Kalarchian MA Steffen KJ Engel SG. Prevalence of alcohol use disorders before and after bariatric surgery. JAMA (2012) 307(23):2516–25. doi: 10.1001/jama.2012.6147 PMC3682834 22710289

[33] Li Z Zhou X Fu W. Vitamin d supplementation for the prevention of vitamin d deficiency after bariatric surgery: A systematic review and meta-analysis. Eur J Clin Nutr (2018) 72(8):1061–70. doi: 10.1038/s41430-017-0059-9 29288249

[34] Monica Petasne N Adriana C Shani S Sosa J. Comprehensive behavioral-motivational nutrition education improves depressive symptoms following bariatric surgery: A randomized, controlled trial of obese Hispanic americans. J Nutr Educ Behav (2013) 45(6):620–6. doi: 10.1016/j.jneb.2013.04.264 23819903

[35] Acuna-Izcaray A Sanchez-Angarita E Plaza V. Quality assessment of asthma clinical practice guidelines: A systematic appraisal. Chest (2013) 144:390–7. doi: 10.1378/chest.12-2005 23450305

