# Supplementary material for: Quality evaluation of metabolic and bariatric surgical guidelines
Source: Front Endocrinol (Lausanne). 2023 Mar 9;14:1118564. doi: 10.3389/fendo.2023.1118564 (PMC10035593; doi:10.3389/fendo.2023.1118564)
Supplement: Supplementary file 1 [file Table_1.docx]

**Table S1 23 items of AGREE II**

| **DOMAIN 1. SCOPE AND PURPOSE** |
| --- |
| Item 1: the overall objective(s) of the guideline is (are) specifically described |
| Item 2: the health question(s) covered by the guideline is (are) specifically described |
| Item 3: the population (patients, public, etc.) to whom the guideline is meant to apply is specifically described |
| **DOMAIN 2. STAKEHOLDER INVOLVEMENT** |
| Item 4: the guideline development group includes individuals from all the relevant professional groups |
| Item 5: the views and preferences of the target population (patients, public, etc.) have been sought |
| Item 6: the target users of the guideline are clearly defined |
| **DOMAIN 3. RIGOR OF DEVELOPMENT** |
| Item 7: systematic methods were used to search for evidence |
| Item 8: the criteria for selecting the evidence are clearly described |
| Item 9: the strengths and limitations of the body of evidence are clearly described |
| Item 10: the methods for formulating the recommendations are clearly described |
| Item 11: the health benefits, side effects, and risks have been considered in formulating the recommendations |
| Item 12: there is an explicit link between the recommendations and the supporting evidence |
| Item 13: the guideline has been externally reviewed by experts prior to its publication |
| Item 14: a procedure for updating the guideline is provided |
| **DOMAIN 4. CLARITY OF PRESENTATION** |
| Item 15: the recommendations are specific and unambiguous |
| Item 16: the different options for management of the condition or health issue are clearly presented |
| Item 17: key recommendations are easily identifiable |
| **DOMAIN 5. APPLICABILITY** |
| Item 18: the guideline describes facilitators and barriers to its application |
| Item 19: the guideline provides advice and/or tools on how the recommendations can be put into practice |
| Item 20: the potential resource implications of applying the recommendations have been considered |
| Item 21: the guideline presents monitoring and/or auditing criteria |
| **DOMAIN 6. EDITORIAL INDEPENDENCE** |
| Item 22: the views of the funding body have not influenced the content of the guideline |
| Item 23: competing interests of guideline development group members have been recorded and addressed |
