# Supplementary material for: Quality evaluation of metabolic and bariatric surgical guidelines
Source: Front Endocrinol (Lausanne). 2023 Mar 9;14:1118564. doi: 10.3389/fendo.2023.1118564 (PMC10035593; doi:10.3389/fendo.2023.1118564)
Supplement: Supplementary file 2 [file Table_2.docx]

**Table S2 Oxford Centre for Evidence-Based Medicine: Levels of Evidence**

| Recommended strength | The quality of evidence | Describe |
| --- | --- | --- |
| A | 1a | Systematic Evaluation (SR) Based on homogeneity of Randomized Controlled Trials (RCTS) |
|  | 1b | Single randomized controlled trial (narrow confidence interval) |
|  | 1c | All or nothing |
| B | 2a | SR (Homogeneity) based on cohort Study |
|  | 2b | Single-cohort studies (including low quality RCTs; for example, <80% follow-up) |
|  | 3a | A homogenous case–control study of SR |
|  | 3b | A single case–control study |
| C | 4 | Case reports (and poor quality cohort studies) |
| D | 5 | Expert opinion or comment |
