# Supplementary material for: Quality evaluation of metabolic and bariatric surgical guidelines
Source: Front Endocrinol (Lausanne). 2023 Mar 9;14:1118564. doi: 10.3389/fendo.2023.1118564 (PMC10035593; doi:10.3389/fendo.2023.1118564)
Supplement: Supplementary file 3 [file Table_3.docx]

**Table S3 Rating of ICCs Evaluation Degree**

| **ICC** | **Degree of Agreement** |
| --- | --- |
| 0.01-0.20 | slight |
| 0.21-0.40 | fair |
| 0.41-0.60 | moderate |
| 0.61-0.80 | high |
| 0.81-1.00 | perfect |

ICC: Intraclass correlation coefficient.
